# Impact of community-based employment on Aboriginal and Torres Strait Islander wellbeing, aspirations, and resilience

**DOI:** 10.1186/s12889-024-17909-z

**Published:** 2024-02-16

**Authors:** Elizabeth Doery, Lata Satyen, Yin Paradies, Graham Gee, John W. Toumbourou

**Affiliations:** 1 School of Psychology, Deakin University, Burwood, Australia; 2 School of Humanities and Social Science, Deakin University, Burwood, Australia; 3 Murdoch Children’s Research Institute, Melbourne, Australia

**Keywords:** Aboriginal and Torres Strait Islander, Employment, Resilience, Wellbeing, Aspirations, Empowerment

## Abstract

**Background:**

This study evaluated a research project that provided employment in an Aboriginal and Torres Strait Islander community-based setting and supported participants to identify and achieve their goals and aspirations. The evaluation examined changes in personal, relationship, community and cultural strengths and resources and explored empowerment and resilience, in terms of promoting wellbeing.

**Methods:**

Ten Aboriginal people employed as life coaches and peer researchers participated in semi-structured interviews and also completed the Aboriginal Resilience and Recovery Questionnaire at the beginning of their employment and 6-months after employment. Interviews with the 10 participants explored changes in their wellbeing, relationships, resilience, opportunity to lead, aspirations, goal setting skills, connection to culture and community, and empowerment.

**Results:**

Participants personal strengths, and cultural and community strengths, sub-scale scores showed improvements across the 6-month period, however these changes were not statistically significant. Using reflexive thematic analysis, we generated five themes including Aspirations; Personal capabilities; Constraints to wellbeing; Community engagement and cultural connection; and Employment facilitators. Overall, participants identified that despite the challenges of their work and the additional challenges posed by the COVID-19 lockdowns, they were able to develop their skills to set and achieve goals. They reported feeling empowered and proud of their work, and engaged more frequently with their communities and culture.

**Conclusions:**

The study outcomes evidence the role of employment in an Aboriginal and Torres Strait Islander community-based project in strengthening wellbeing, enhancing resilience, and supporting participants to advance their personal goals and aspirations. These findings reinforce the importance of supporting the aspirations and employment of Aboriginal and Torres Strait Islander Peoples through employment.

**Supplementary Information:**

The online version contains supplementary material available at 10.1186/s12889-024-17909-z.

## Background

Ongoing colonial practices and policies continue to have negative impacts for many Indigenous Peoples globally [1]. The distress caused by these historical and contemporary practices are linked to poorer mental and physical health outcomes, lower education and employment rates, and greater incidents of incarceration rates among Aboriginal and Torres Strait Islander Peoples [2]. Efforts to engage effectively and genuinely with communities and to place greater emphasis on Aboriginal and Torres Strait Islander voices have resulted in significant health and wellbeing gains [3, 4]. However, as scholars including Paradies highlight, substantial decolonization efforts are still required [1]. This includes working alongside communities to develop effective decolonizing strategies and supporting the self-determination, wellbeing, and capacity-building of Aboriginal and Torres Strait Islander Peoples. Steps towards decolonization include implementing strength and community-based approaches [5], considering non-Western realities and knowledge systems [6], acknowledging existing power dynamics, prioritizing the voices of community [7], and approaching research with reflexivity [8, 9].

### The Social and Emotional Wellbeing Framework

Aboriginal and Torres Strait Islander conceptualization of Social and Emotional Wellbeing (SEWB) was articulated at a policy level in 2004 as part of the National Strategic Framework for Aboriginal and Torres Strait Islander wellbeing [10]. This helped to formally recognize and acknowledge Aboriginal and Torres Strait Islander conceptualizations of health and wellbeing [11]. The framework, and its subsequent iterations offers health workers, researchers, organisations, government agencies, and policymakers a guide to support and strengthen the SEWB of Aboriginal and Torres Strait Islander Peoples [12].

The framework draws on the definition of Social and Emotional Wellbeing, which encompasses a holistic view of mental health and recognizes that communities’ social, emotional, and cultural wellbeing impacts the individual [13]. SEWB within this context includes connections to spirituality, community, language, kinship, culture, Country, family, and ancestry in addition to social, political and historical determinants [2]. Self-determination, resilience, a sense of identity, and empowerment are also domains central to conceptualizing SEWB, and importantly there is diversity with regards to how these domains are manifest across cultural groups [12, 14].

### Cultural and community connection

Two determinants of wellbeing that are a focus of the current study are: connection to culture and to community. Research has demonstrated the importance of culture and community connection for the wellbeing of Aboriginal and Torres Strait Islander Peoples [9, 15, 16]. For example, cultural engagement has been found to be associated with reductions in suicide- and alcohol-related risk behaviors [17] and enhancing positive behaviors [18]. Dockery has argued that health and wellbeing outcomes can be better promoted through building strong affiliations and cultural engagement [15]. Furthermore, Doery and colleagues found that community engagement is positively related to young adult development among Indigenous youth [16]. Similarly, Shepherd and colleagues found that cultural engagement was significantly related to non-recidivism among adult Indigenous Peoples in custody [19].

One approach for enhancing the opportunity for Aboriginal and Torres Strait Islander Peoples to engage with their communities and culture is through employment. For example, Hill and colleagues summarized some of the key benefits of employment in Indigenous land management programs [20]. These included enhanced health and wellbeing among those involved, environmental benefits, intergenerational transfer of knowledge, cultural tourism, increased capacity, improved relationships, and the production of food sources. Similarly, Kingsley and colleagues highlight that combining cultural activities with education and employment could be a key strategy to improve health outcomes [21]. Building on this existing literature, the current study explored how community and cultural engagement can be enhanced through employment in a community-based project.

### Resilience and empowerment

The terms *resilience* and *empowerment* are frequently cited within Aboriginal research pertaining to SEWB. Broadly, resilience has been conceptualized as the process of overcoming significant adversity, which for many Aboriginal and Torres Strait Islander Peoples, includes the past and ongoing impact of colonization and intergenerational trauma [22]. For many Aboriginal and Torres Strait Islander People, resilience comes in the form of family, culture, community, kinship, Country, ancestry, and personal skills [22–24]. Empowerment relates to having a greater sense of control and developing an awareness of one’s skills, knowledge, and confidence [3]. Some research has found that resilience and a sense of empowerment can be enhanced through Aboriginal and/or Torres Strait Islander-designed programs, projects, and strategies. One such approach is the National Empowerment Project which is a community-based project that enhanced healing, leadership, capacity, and capability of whole communities [3]. Similarly, Gee and colleagues found that Victorian Aboriginal-designed programs with a focus on empowerment and wellbeing were associated with increases in access to strengths and resources among participating community members [22]. Such studies demonstrate the importance of engagement with projects that focus on promoting resilience and a sense of empowerment among Aboriginal and Torres Strait Islander communities.

### Goals and aspirations

There are a range of both barriers and protective factors to achieving life goals. Barriers can include financial strain, lack of support, competing priorities and psychological factors (e.g., self-actualization and personal satisfaction) [25, 26]. Typically, protective factors tend to encompass both external resources (e.g., family support) and internal resources (e.g., determination). For Aboriginal and Torres Strait Islander communities, barriers might include social injustice associated with colonization, discrimination, and racism. For example, Browne-Yung and colleagues explored the experiences of social capital among Aboriginal peoples and found that they experienced exclusion and racism in the workplace and that their identity likely impacted their income and employment goals [27].

In contrast, protective factors tend to encompass both external resources (e.g., family support) and internal resources (e.g., determination) [25]. Research conducted with Aboriginal and Torres Strait Islander Peoples has shown some protective factors including cultural engagement and support from family, friends, and mentors [28, 29]. The current study explored the potential role of cultural engagement, support, and employment in community-based research as protective factors for achieving life goals and aspirations. We also explored participants’ specific goals and aspirations, the barriers they faced in achieving these goals, and strategies to further promote goal and aspiration attainment.

### Employment and wellbeing

Due to persistent social and economic inequalities experienced by many Aboriginal and Torres Strait Islander Peoples, scholars continue to explore pathways to greater equality [9]. Osborne and colleagues reviewed the role of employment and income among Aboriginal and Torres Strait Islander Peoples to enhance social and economic outcomes [30]. While noting the complex relationship between employment, income, and wellbeing, the authors argued that employment should lead to higher income, which in turn provides access to greater resources for maintaining or strengthening wellbeing [30]. National surveys have found that Aboriginal and Torres Strait Islander Peoples who are employed are also more likely to report higher rates of happiness than those who are unemployed [31]. Similarly, international literature has highlighted the negative impact of unemployment on happiness and further suggests the distress that unemployment can cause [32].

There is also research that suggests avenues to promote wellbeing within the workplace. For example, Dockery and Milsom reported that when employment is combined with a support or mentorship program, it is more likely to foster improved wellbeing and self-determination [33]. This suggests that, although enhancing employment opportunities holds value, it is equally crucial to establish a supportive and inclusive environment and foster a strong sense of cultural engagement and identity, to achieve enhanced wellbeing outcomes. Scholars further propose that strategies like prioritizing the voices of Aboriginal and Torres Strait Islander Peoples and recognizing cultural backgrounds may be more effective in promoting wellbeing [33, 34]. Consequently, integrating both employment and a strong connection with community and culture through participation in community-based research emerges as a particularly significant strategy for enhancing wellbeing.

There is also international literature that has explored the relationship between employment and mental health among the general population. For example, the World Health Organisation (WHO) states that there are different workplace conditions that can enhance or reduce our sense of mental wellbeing and quality of life both within and outside the workplace [35]. Further work proposes that while there are harms of working too little or too much working in moderation can be correlated with greater happiness [36]. However, this literature also notes the substantial individual variation that exists and the impact of the workplace environment. While considering this literature, as Blustein points out, it is also important to acknowledge that there are many social barriers that people face preventing them from working or reducing access to work [37]. Together with the research focused on Aboriginal and Torres Strait Islander communities, this existing literature highlights the complex relationship between mental wellbeing and employment. It also suggests that there are strategies, approaches, and interventions that can be implemented within workplaces to greater sense of wellbeing.

Aboriginal and Torres Strait Islander community members are increasingly being employed in community-based research. For example, Kelly and colleagues explored the experiences of eight Aboriginal and Torres Strait Islander community-based researchers who were employed to deliver community research in remote and rural communities [34]. As part of their role, the community researchers received training, mentoring, and support from experienced researchers. While the researchers had distinct experiences, a common reflection was the importance they placed on supporting their community and achieving better health outcomes for community members [34]. Haynes and colleagues also examined outcomes for Aboriginal community researchers, including changes in empowerment, power relations and policies/practice [7]. They found an increase in community capacity and a desire among community researchers to increase their knowledge and skills within a research context. Community researchers also faced challenges, including conflicting cultural demands and feeling a sense of inadequacy [7]. There is a need for more research investigating the wellbeing impacts of employment in research among Aboriginal and Torres Strait Islander community members. The current study investigated employment and its relationship with participants’ cultural and community engagement, aspirations, and wellbeing.

The current authors acknowledge the importance of implementing ethically and culturally safe research. Therefore, to make a concerted effort to achieve this, we worked alongside Aboriginal and Torres Strait Islander researchers and the participants and referred to existing guidelines [2, 38]. This involved implementing principles of cultural safety, awareness, competency, and sensitivity. Following Heckenberg’s recommendation, we recognized and respected differences across and within cultures, developed our understanding of these different cultural perspectives, and developed our skills to connect effectively with members from diverse communities [38]. As is detailed in our previous paper [39], we strived to implement these principles by attending cultural training, engaging deeply and genuinely with the participants, reflecting on our research practices, and engaging with community members, the literature, other researchers, and working in partnership with Indigenous researchers.

## The current study

Given the paucity of research within this field and the importance of the genuine inclusion and employment of Aboriginal and Torres Strait Islander Peoples, the current study explored the experiences and wellbeing of Aboriginal and Torres Strait Islander life coaches (LCs) and peer researchers (PRs). The LCs and PRs were recruited to participate in the More Than A landlord (MTAL) research project led by the University of Melbourne and Aboriginal Housing Victoria (AHV). MTAL is an ongoing community-based project that aims to evaluate the impact of a life coaching program on tenants of AHV. A key element of this project is the inclusion of Aboriginal and Torres Strait Islander community members as PRs and LCs [40]. As part of this project, the primary roles of the PRs were participant recruitment, baseline data collection, and to act as the conduits between the research team and the wider Victorian Aboriginal community [39, 40]. Many of the PRs were new to employment and were tenants of AHV themselves. Their dual roles as community members and peer researchers afforded them a profound understanding of the community dynamics, and their contributions not only reinforced their own connection with the community but also contributed to community capacity building [39]. The cohort of LCs included both newly employed staff and existing life coaching staff. Among those newly employed, backgrounds in life coaching, wellbeing, or community development were prevalent [36]. The primary role of the LCs was to provide culturally relevant life coaching to AHV tenants involved in the MTAL study [39, 40]. Further detail on MTAL can be found in our colleagues’ paper [40].

The primary aim of the current mixed-methods study was to evaluate the impact of employment in this community-based project on the lives of Aboriginal and Torres Strait Islander peer researchers and life coaches. Specifically, we collected predominately qualitative data, with the aim to explore participants’ experiences of goal setting and attainment; cultural and community connection; empowerment; leadership; overall wellbeing; relationships; and resilience, and the impact of employment on these factors. This qualitative data was supported by a small-scall quantitative component, which aimed to explore one research question: Do Aboriginal and Torres Strait Islander life coaches and peer researchers experience changes in personal, relationship, community and cultural strengths and resources after 6 months of employment in a research project? We hypothesised that perceived personal, relationship, community, and cultural strengths and resources would increase from baseline to 6 months among the PRs and LCs. We utilised a strength-based approach that included engaging in deep and respectful conversations with the participants, focusing on positive wellbeing, determining strategies to improve wellbeing outcomes, and highlighting and privileging participant voices [39]. Researchers E.D and L.S also attended the peer researcher and life coaching training. Details of this training can be found in our previous paper [39]. As members of the co-design team (which included community members and academics across multiple institutes), researchers G.G and Y.P were responsible for formulating the overarching project objectives in conjunction with members of the Aboriginal community.

## Method

### Design

To assess the study outcomes, a mixed-methods longitudinal design was implemented. The study received approval from the University of Melbourne Human Research Ethics Committee (ethics ID 2020–13,595–13162-4) and from the first author’s Institutional Human Research Ethics Committee (2021-013). A Memorandum of Understanding was also signed between Victorian Aboriginal Health Services and the first author’s institution to use the Aboriginal Resilience and Recovery Questionnaire.

For the qualitative data collection, participants completed a 1-hour semi-structured interview (see appendices A-E). Throughout the collection and analysis of the qualitative data, all research members considered the role of subjectivity, engaging in reflexivity by considering our biases, beliefs, backgrounds, experiences, knowledges, and limitations [24, 41]. The research team included both Indigenous and non-Indigenous researchers who engaged in regular discussions related to appropriate research methodology and analysis at all stages of the project. In the development and implementation of this study, the research team also worked alongside a team of Indigenous and non-Indigenous researchers at the University of Melbourne and staff at AHV. The authors adhered to a constructionist epistemological approach in developing the interview questions and in interpreting the participant responses. In this way, we engaged with participants to construct their own meaning and reality of a single phenomenon (i.e., working as either a peer researcher or life coach) and have their own perspective regarding this experience [42]. This approach also acknowledged that an individual’s cultural, historical, and social perspectives impact the way they understand their world and engage with others in their community [42, 43].

For the quantitative data collection participants completed a 63-question survey (see appendix F), which included multiple items from the Aboriginal Resilience and Recovery Questionnaire (ARRQ) [44, 45] in addition to demographic questions. The ARRQ is designed to be a strength-based measure that assesses access to personal, relationship, community and cultural strengths and resources among Aboriginal and Torres Strait Islander Peoples [39, 44]. Baseline data was collected between March and November 2021 and 6-month data was collected between September 2021 and May 2022. Further detail regarding the study design can be found in our protocol paper [39].

### Participants

Eligible participants were Aboriginal and Torres Strait Islander LCs and PRs currently employed by AHV to work on the MTAL project. Participation in the study was voluntary and did not impact employment at AHV. To reimburse their time, all participants received a $35 shopping voucher for each session in which they participated. Recruitment into the current study took place at a two-week training program attended by all eligible participants and two researchers (L.S and E.D). The training provided all staff and the attending researchers with an opportunity to develop their research and coaching skills, their understanding of self-care strategies, their communication skills, and their cultural knowledge and awareness. One researcher (E.D) also presented an overview of the current study providing prospective participants with the opportunity to gain a detailed understanding of the project, to ask questions, and to provide feedback before deciding whether to be involved. Baseline survey data was collected on the last day of training. Additional information regarding the recruitment of the PRs and LCs and the training can be found in our colleagues’ papers [39, 40].

Eleven PRs and LCs employed by AHV attended the training and were considered eligible to participate in the current study. Of those eligible to participate, at baseline 11 people participated in the quantitative data collection and 10 in the qualitative data collection. Due to personal reasons one participant did not continue working as a PR following the training and therefore only completed the baseline quantitative data collection and was not included in the final data analysis. From the 10 people who completed the baseline quantitative and qualitative data collection, one person did not complete the 6-month quantitative or qualitative data collection due to personal reasons. For this participant, their baseline qualitative data was maintained but their baseline quantitative data was not included in the final data analysis. Overall, we present qualitative data from 10 participants (five PRs and five LCs) at baseline and nine participants (four PRs and five LCs) at 6 months. We also present quantitative data from nine participants (five LCS and four PRs) collected at both baseline and 6 months. The demographic information in Table 1 presents data collected with 10 participants at baseline.

**Table 1:** Demographic characteristics measured at baseline

Participant number	Job title	Cultural group	Age	Marital status	Living arrangement	Highest level of education	Sources of income	Currently looking for work	Problems getting a job
Participant 1	Peer researcher	Aboriginal, Torres Strait Islander	17	Single	Staying with friends temporarily	Certificate I/11	Paid employment	Yes	Transport, too far to travel
Participant 2	Peer researcher	Aboriginal, First Nations, First Peoples	21	In a relationship, not living together	Owned house/apartment	Advanced Diploma	Paid employment/ Government allowance	No	Doesn’t have a drivers license
Participant 3	Peer researcher	Aboriginal, Torres Strait Islander	21	Single	Rented house/apartment	Certificate I/II	Prefer not to answer	Yes	No jobs in local area
Participant 4	Peer researcher	Aboriginal	35	Married	Refuge/ temporary accommodation	Post-graduate degree	Government allowance	No	Criminal record
Participant 5*	Peer researcher	Aboriginal	41	In a relationship, living together	Rented house/apartment	Advanced Diploma	Paid employment, Government allowance	Yes	Ill health/disability
Participant 6	Life coach	Aboriginal, Torres Strait Islander	50	Married	Rented house/apartment	Certificate III/IV	Paid employment	No	Insufficient training
Participant 7	Life coach	Aboriginal	32	Married	Rented house/apartment	Certificate III/IV	Paid employment, Government allowance	No	No job to match skills
Participant 8	Life coach	Aboriginal, First Nations, First Peoples	25	In a relationship, not living together	Rented house/apartment	Bachelor degree	Paid employment	No	None
Participant 9	Life coach	Aboriginal	38	Single	Rented house/apartment	Certificate III/IV	Paid employment	No	None
Participant 10	Life coach	First Nations, First Peoples	42	Married	Rented house/apartment	Bachelor Degree	Government allowance	No	None

* Participant did not participate in 6-month data collection

### Survey

The survey, which comprised of the ARRQ and three additional demographic questions, took approximately 15 min to complete online via Qualtrics. The survey also included a detailed summary of the study, a consent form, and Plain Language Statement. Participants were required to read the Plain Language Statement and sign the consent form before partaking in both the survey and interview. The ARRQ is a 60-item measure that was developed at the Victorian Aboriginal Health Service (VAHS) to assess personal, cultural, community, and relationship strengths and resources found to be associated with a range of wellbeing outcomes [22, 44, 45]. Participant responses are rated on a 5-point likert scale where 1 = Not at all, 2 = A little, 3 = Somewhat, 4 = A fair bit, and 5 = A lot. The ARRQ comprises two key subscales, the personal strengths and resources subscale and the cultural, community and relationship strengths and resources subscale. An example of a question from the personal strengths and resources subscale is “Despite any bad experiences in the past I am able to trust most people”. Gee demonstrated that the measure has high internal consistency with a Cronbach’s alpha value of 0.93 for the total ARRQ measure [44].

### Interviews

Semi-structured interviews were conducted in English and were either completed face-to-face or online (using the platform Zoom) when COVID-19 restrictions did not permit face-face interviews. All interviews were conducted with one researcher (E.D), audio and manually recorded (with permission from each participant prior to the interview). These notes and recordings were securely stored at the first author’s institution in a locked cabinet and on a password protected computer. Data was collected and stored in compliance with the Victorian and Commonwealth privacy law. Examples of demographic questions (see Table 1) included participants’ status of Indigeneity, age, and any difficulties experienced in the past whilst finding a job. During the baseline interviews, participants were asked to set health, employment, family, education, finance, and housing goals and describe how they expected involvement in the community-based project to impact their ability to achieve these set goals, wellbeing, cultural and community connection, relationships, leadership opportunities, sense of empowerment and resilience. The 6-month interviews provided participants with an opportunity to reflect on these same factors and how they might have changed over the course of 6-months working at AHV. During both the baseline and 6-month interview, participants were also asked to reflect on any relevant challenges. A unique identifier was given to all participants to match their data collected at the two time points and to provide confidentiality.

## Analysis

### Quantitative data

Paired Samples *t* Tests were conducted to examine changes in participants’ score on the ARRQ from baseline to 6-months using the IBM statistical software package SPSS [46]. Specifically, two separate Paired Samples *t* tests were run examining changes in participants’ reported access to personal strengths and resources and relationship-community-cultural strengths.

### Qualitative data

Audio recordings were imported into Microsoft Excel for analysis purposes by the first author [39]. Given its previous use to analyze data collected with First Nations communities, thematic analysis was implemented [24, 47]. As part of the data interpretation process, the authors also engaged in reflexivity, drawing on the principles of reflexive thematic analysis. There are three approaches or subtypes of thematic analysis, reflexive thematic analysis being one of which. This approach encourages researchers to consider their own biases and assumptions when interpreting data. As a research team that includes both Indigenous and non-Indigenous academics, we concluded that engaging in reflexivity is an important step in the analysis process. This reflexive process involved considering our own social position (i.e., class, race, ethnicity), identities, and knowledge, and the impact of these on how data is interpreted [48]. However, while considering personal biases, opinions, and attitudes and the impact of these on the interpretation of the data (inductive coding) the researchers also aimed to capture participants’ voices objectively and accurately (deductive coding) [48]. To complete the reflexive thematic analysis and identify patterns within the research, the 19 interview transcripts were read a minimum of three times and phrases which seemed meaningful in responding to the research aims were highlighted. These phrases were collated in a table to assist the coding process. Codes were then arranged into themes. As per our Protocol, theme development was informed by Aboriginal knowledge experts who have expertise in formulating culturally relevant themes [35]. These experts, who have doctoral qualifications and have published extensively in the field, were part of the implementation of the study design and contributed to the formulation of each theme. All five co-authors were involved in developing the codes and themes.

## Results

The baseline sample consisted of five PRs and five LCs aged between 17 and 51 years. The 6-month assessments were completed by four PRs and five LCs. The demographic characteristics of all participants collected at baseline are presented in Table 1. All participants self-identified as one or more of Aboriginal, Torres Strait Islander, First Nations, or First Peoples. There were several different clan groups represented across the participant group including Wuthathi, Yorta Yorta, Bangerang, and Noongar. All participants completed a minimum of Certificate I/II. Within Australia, certificate training provides workplace skills ranging from more basic or general skills at Certificate I (Level 1) to more specialized or complex skills at Certificate IV (Level 4). The highest level of education that participants had achieved was a post-graduate degree. Most participants lived in either a rented house or apartment (60%). While three participants (30%) experienced no difficulties in getting a job, others experienced some difficulties including ill health/disability, insufficient training, and the lack of suitable jobs to match their skills.

### Quantitative results

Descriptive statistics of baseline and 6-month outcomes among nine participants are presented in Table 2. Two separate Paired Samples *t* tests were run to compare the baseline scores (113.29) on the *Personal Strengths and Resources* scale with the 6-month scores (128.56) and the baseline scores (90.46) on the *Relationship-Community-Cultural strengths* scale with the 6-month scores (96.33). On average participants’ 6-month *Personal Strengths and Resources* scores were 15.27 points higher than their baseline scores, 95% CI [-0.62-12.36]. However, the difference was not statistically significant, *t*(9) = 2.08, *p* =.07 Cohen’s *d* for this test was 0.69 which is considered a moderate effect size. On average participants’ 6-month *Relationship-Community-Cultural strengths* scores were 5.87 points higher than their baseline scores, 95% CI [2.17–32.70]. However, the difference was not statistically significant, *t*(9) = 2.02, *p* =.08. Cohen’s *d* for this test was 1.02 which is considered a large effect size.

**Table 2:** Participant Personal Strengths, and Relationship–community–cultural Strengths scores at baseline and 6-months

Outcomes	Baseline and 6-month comparisons						
	Baseline M (SD)	6-month M (SD)	Mean Difference	95% CI	*t*	*p*	Cohen’s *d*
Personal strengths (*n* = 9)	113.29 (17.75)	128.56 (11.70)	15.27	-2.17-32.70	2.02	0.08	1.02
Relationship-Community-Cultural strengths (*n* = 9)	90.46 (8.00)	95.33 (6.06)	5.87	-0.62-12.36	2.08	0.07	0.69

### Qualitative results

An initial set of 62 codes was developed, which was then modified and collapsed to 46 codes following discussions between researchers E.D and L.S. To further refine codes and develop themes, the remaining team were consulted (Y.P., J.T., and G.G.) until a consensus was reached [39]. This process resulted in the generation of five themes. These themes were Aspirations; Personal capabilities; Constraints to wellbeing; Community engagement and cultural connection; and Employment facilitators. Table 3 provides a summary of the themes and codes and includes relevant quotes, which are elaborated in the following sections.

**Table 3:** Qualitative results demonstrating the impact of employment in a community-based project

Themes	Data collection time point				
	Baseline			6-months	
	Codes	Quotes		Codes	Quotes
1. Aspirations	**1.1 Physical Health**	*“I want to start going to the gym”* (PR) “*Start eating food that energizes me”* (PR) *“Want to be healthier Yeah, I just want to feel healthier”* (LC)		**1.6 Goal tracking**	*“I would say I have achieved these [goals]”* (LC) *“still the same it got good then it got bad”* (LC) *“I feel like I’ve accomplished a lot”* (PR) *“I was really happy when I got the car…I was that excited when I got it”* (PR) *“no, still working on them I think last time it was the house…still doing that”* (LC) *“So I haven’t exactly made like huge jumps or steps or anything…but made a slow start”* (PR) *“I actually have an interview”* (PR) *“on the way yea, definitely…goals shouldn’t finish”* (LC) *“setting big goals”* (LC))
	**1.2 Employment and education**	*“want to work with my mob and community”* (PR) *“starting my own business”* (LC) *“I’m one of those people that won’t stop learning”* (LC)			
	**1.3 Family and housing**	*“spend time with my family and enjoy it”* (LC) *“buy a property in Melbourne”* (LC) *“just comfortable with accommodation”* (PR) *“bring my family back together”* (LC)			
	**1.4 Financial**	*“if we want to talk financial wellbeing, which it definitely has. Yea because I am making decent money”* (PR) *“So me personally, I’ve always wanted to be able to be financially independent… having financial independence myself, is empowering”* (LC)			
	**1.5 Identifying skills and strategies to achieve goals**	*“you realise that you actually have goals and aspirations”* (LC) *“the more I physically talk about it, the more I’m going to want to do it”* (PR) *“That’s a goal for six months when I get asked that question…write it on a piece of paper, put it on a mirror and look at it every day”* (LC)		Not applicable	
	Not applicable			**1.7 Reflection of goals**	*“when I was last speaking to you about it…I’m looking at it from a different point”* (LC*)* *“mostly thinking about like when lockdown ends like, what can I do to move towards those goals”* (PR) *“I’ve been thinking about the goals over the last few weeks”* (PR)
	Not applicable			**1.8 Structuring new goals**	“*helped me structure and plan my goals better for myself”* (LC) *“my mindset really…I’m grateful that I have a job…really strive for those and obtain those goals…you can actually do it”* (LC) *“I set myself goals everyday”* (LC)
2. Personal capabilities	**2.1 Importance of confidence**	*“professional training so they can feel more confident”* (LC) *“do all those things and feel confident about it”* (LC) *“giving me more confidence to speak”* (PR)		**2.8 Increased Confidence**	*“very confident now that what I do for the organisation”* (LC) *“So at first it was a bit challenging…eventually I was able to overcome that”* (PR) *“my confidence in general is going up… not even just with work, just talking to people in general or anything I do”* (PR)
	**2.2 Internal motivation**	*“don’t give up…keep on trying”* (LC) *“hold yourself accountable has really been amazing”* (LC) *“I probably wouldn’t be motivated”* (PR)		**2.9 Internal Motivation**	*“made me want to work harder”* (PR) *“I’m stubborn. I don’t give up…I have to be able to look myself in the mirror and say I have done everything”* (LC) *“it’s me doing it for myself now”* (PR)
	**2.3 Sense of pride**	*“it was moving fast and I kept up” (*PR) *“lead by example*” (LC) *“I’m proud of myself…sometimes I feel like it’s a struggle but I’m doing it*” (PR)		**2.10 Sense of pride and self-worth**	*“real proud of myself”* (PR) *“I’m celebrating it…my mindset around things has changed”* (LC) *“Believe it has changed who I am”* (LC) *“I am capable of this”* (LC)
	**2.4 Empowerment**	*“gives you a bit of empowerment and responsibility”* (PR) *“makes me feel empowered by believing in myself”* (PR) *“I’ve always advocated for myself always been very vocal about what I need”* (LC)		**2.11 Empowerment**	*“feel empowered”* (PR) *“empowered to want to help people”* (LC) *“I know what it [empowerment] looks like, when you watch somebody find it. When I watch my clients find it, that’s empowering for me”* (LC)
	**2.5 Skill development**	*“I definitely think maybe there’s some skills…that I can learn from this role and carry onto future roles”* (PR) *“I’ve gotten better at talking to people”* (PR) *“I’ve got the position and got the skills, reinforce that the skills I have”* (LC)		**2.12 Skill development**	“*I’ve learnt new things”* (PR) *“it used to give me a headache but now I sorta know what to do”* (PR) *“I’m definitely getting skills that I can use”* (LC)
	**2.6 Resilience**	*“probably resilience, because it’s just I look at other people’s lives and it’s like actually I’m not going through it that hard as, as hard as others” (PR)* “*gonna make me stronger”* (LC*)* *“been very fortunate in what I’ve gone through has helped shape me”* (LC)		**2.13 Resilience**	*“mentally, physically yea, I’m not having days where I’m excessively down”* (LC*)* *“I’m not falling into tears and a mess”* (LC) *“yea I am quite resilient, particularly through covid”* (LC) *“it made me a stronger person and made me more resilient”* (PR)
	**2.7 Sense of gratitude**	*“you actually recognize how, how blessed your life has been regardless whether you thought it was blessed at the time and there’s days that you just think the whole world sucks”* (LC) “*So it makes me become more grateful”* (PR) *“it’ll make me feel very for fortuitous”* (LC)		**2.14 Sense of gratitude**	*“it’s more of an eye opener…there are a lot of people in need…puts yourself in perspective”* (LC) *“really opened my eyes to how bad it can be and how awful some of these situations are”* (PR)
	Not applicable			**2.15 Seeking help**	*“I would have never asked for help”* (LC) “*I found that was very empowering. Like it was not that bad. Asking for help. Yea. I did it and it wasn’t that bad”* (LC) *“…helped me through how I can save slowly”* (PR)
	Not applicable			**2.16 Leadership**	*“I like being given more responsibility”* (LC) *“I’m not really at the right age to be leading much”* (PR) *“I have an assistant now and I have never had that before”* (PR)
3. Constraints to wellbeing	**3.1 Cultural and community responsibilities**	*“it’s a lot for one individual to take home”* (LC) *“with tenants trauma and how I process that” (*LC) *“it’s dredging up the past”* (PR)		**3.5 Cultural and community responsibilities**	*“hearing these situations and not actually being able to help or not actually having any resources”* (LC) *“I think like the client facing was just a lot of emotional capacity”* (LC) *“less opportunities to like go out…never done any I guess cultural activities…had to be cancelled”* (PR)
	**3.2 Impact of colonization**	*“to be honest, no not really I haven’t really spent too much of my life talking or discussing it”* (PR) *“missing a part of myself and my identity. Someone took that away and that sucks”* (LC) *“that transgenerational trauma and try to stop it”* (PR)		**3.6 Impact of colonization**	*“being in Melbourne, I feel quite removed from, from the background that I grew up in”* (LC) *“once I’ve got my COA [certificate of Aboriginality] I will feel a lot more comfortable”* (LC)
	**3.3 Level of support**	*“we need to get more professional debriefing counselling in place”* (LC) *“I could do with some help with that”* (PR) *“having our own life coach, which I think makes so much sense…work on your own goals”* (LC)		**3.7 Level of support**	“*feel like that I’m just doing it on my own”* (LC) “*just like support in this program would have been so cool*” (LC) *“when you’re by yourself, its different. So I’m just not used to it”* (PR)
	Not applicable			**3.8 Workplace/** **employment challenges**	*“we couldn’t really work to our full extent…has been really hard”* (LC) *“personally, I think they could have gave me more work to do”* (PR) *“would always frustrate me…this isn’t my role…this whole work I’m doing isn’t what I came into”* (LC)
	**3.4 Poor emotional wellbeing**	*“Personally, it couldn’t have come at a better time for me”* (LC) *“I think with my mental health I just overthink too much”* (PR)		**3.9 Poor emotional wellbeing**	*“It did really impact me in a heavy way”* (LC) *“it did impact on my mental and my well being a little bit, to be honest so it was just facing these challenges head on”* (PR)
4. Community engagement and cultural connection	**4.1 Domains of community and culture that are important to participants**	“*NAIDOC march”* (LC) *“care for country”* (LC) *“going back to country”* (LC) *“think it’s probably important that I learn”* (PR) *“very important to know who you are and where you come from”* (PR)		Not applicable	
	**4.2 Understanding community needs**	*“seeing them do it is enough”* (LC) *“just mob working with mob to help them achieve whatever they want”* (LC)		**4.5 Supporting community needs**	*“I can see the difference…she just got a job. And this all started from me”* (LC) *“help community and be part of this community”* (LC) *“I enjoy actually being able to, to help the tenants”* (PR)
	**4.3 Benefit of community interaction**	*“It’ll just make the bond stronger, more in depth”* (LC) *“they’re…here to share all that knowledge so I love going out there and then when they see a young fella coming in through the door oh my god I love a yarn”* (PR)		**4.6 Benefit of community interaction**	*“having a casual conversation on the phone while you’re doing the survey”* (PR) *“the whole time she was doing the survey she was just telling me stories”* (PR) *“you’re building rapport”* (LC)
	**4.4 Understanding community and culture**	*“would have no idea about these events were happening but because I work here it’s kind of like opening doors to going to events and ceremonies”* (PR) *“this job has actually helped me more, to be connected”* (PR) *“Not as strong as I’d like to be but yeah, we’re getting there”* (LC)		**4.7 Growth in community and cultural understanding**	*“I’ve definitely learned a lot more about m Aboriginal side of my, my family”* (PR) *“it’s been nice being able to learn more about the Aboriginal community here in Melbourne”* (PR) “*I’ve started asking a little bit more questions about like… my tribe and stuff and where I came from”* (PR)
	Not applicable			**4.8 Sense of community at work**	*“it helped having support from work because then that was one less stress”* (LC) *“everyone’s always in a good mood and everyone’s friendly to each other”* (PR) *“team was so beautiful and that support there”* (LC)
5. Employment facilitators	**5.1 Career opportunities**	*“other Indigenous organisations, they can see that I’ve been working with Aboriginal Housing and then that could help”* (PR) *“this job already had, like, it’s led me on to other jobs…so yea definitely set me up for, for other jobs”* (PR) *“as for like further in career…I haven’t really thought about it, because I haven’t really stepped into what this role is like yet”* (LC)		**5.3 Career opportunities**	*“getting your foot in the door, working with community”* (PR) *“I’m my own boss, so excited…I’m excited for that challenge*” (LC) *“Yeah, definitely helps [to get current job]”* (PR)
	Not applicable			**5.4 Realization of future potential**	*“getting this new job, which is full-time so I wouldn’t have that if I didn’t have the role I did at AHV, so yea basically empowered”* (PR) *“there is no way I probably would have done it or even considered it without full-time employment…the security of it as well”* (LC)
	**5.2 Expansion of life coach program**	*“actually like to see this program go further. I’d like to be able to help do that”* (LC) *“being able to train more people, get the vision that I have in my head to…grow in the organization… in the next five years, we’ll have our own department”* (LC)		Not applicable	
	Not applicable			**5.5 Inspiration for further study/career planning**	*“I want to go back to university”* (LC) *“I’ve even contemplated some research study…maybe something in Indigenous research”* (LC)

#### Theme 1: aspirations

During the baseline interviews, the PRs and LCs were asked about their goals related to their physical health, finances, employment, education, housing, and family. Participants spoke about how their physical health (Table 3, code 1.1) was important to them and set goals ranging from eating healthier, exercising more, to generally feeling healthier in themselves. When asked about education and employment goals (code 1.2) participants tended to view employment as a greater priority to them compared to education, discussing how this role may assist their future employment endeavors. Participants either set more specific employment goals such as starting a business or broader goals such as working for community. Family and housing goals (code 1.3) were predominately discussed together. For example, participants spoke about buying a home for their family or wanting to bring the family back together under one house. However, both goals were also spoken about separately by some participants. For example, one participant wanted to “*spend time with my family and enjoy it”*. Financial goals (code 1.4) were a key focus for many participants. Participants spoke about financial independence and how having a consistent income would provide them a sense of security and comfort. For example, one participant stated that *“It’s good to get paid weekly”* and that they just wanted to *“be comfortable.”* Finally, during the baseline interviews, participants discussed whether they had identified the skills and strategies to achieve their goals (code 1.5). If they did have the skills, some participants spoke about what those skills were (e.g., talking to other people about their goals) or if they did not have the skills, they discussed how they might develop them.

During the 6-month interview, participants reflected on the goals they had achieved (codes 1.6–1.8) since the baseline interview. Participants elaborated on specific goals they had achieved or discussed the goal setting skills they had attained. Working for this community-based project and being interviewed for this study provided participants with the opportunity to set themselves goals, reflect on these goals, and celebrate the goals they had achieved. Every participant had achieved at least one of the goals they set out at baseline and they all enthused in the benefit of setting goals. Where some LCs and PRs explained how they made a renewed effort to achieve more complex goals, others reiterated on having a greater understanding of their limits and the need to set more realistic goals. For example, one life coach said that they were *“on track but with more realistic goals now that I’ve sat down and really thought about it”.* Overall, most participants felt proud of their accomplishments and felt they could achieve more goals than they had initially set.

#### Theme 2: personal capabilities

The PRs and LCs identified several personal capabilities that they were eager to develop or felt they already had developed prior to commencing work. These capabilities included confidence (codes 2.1, 2.8), motivation (2.2, 2.9), pride (2.3), self-worth (2.10), help-seeking behaviour (2.15), empowerment (2.4, 2.11), resilience (2.6, 2.13), sense of gratitude (2.7, 2.14), leadership (2.16), and their overall professional skills (2.5, 2.12). During the baseline interviews, most participants acknowledged the importance of these capabilities, both to fulfill their role and more generally in their lives. For example, one LC expressed that *“you need that strength and resilience”* for this job. Most participants also expressed feeling motivated, grateful, and proud. One PR stated that *“it really did actually opened up my eyes a little bit more to my heart as well to be honest”.* This participant acknowledged how important it is to work with their community and the impact that this has on your sense of gratitude.

Changes in personal capabilities across the 6 months that were most evident were confidence, leadership, self-worth, pride, professional skills, and help-seeking behaviour. For example, most PRs and LCs explained how they had become more confident both in themselves and at work within 6 months. Similarly, the PRS and LCs were aware of how much their sense of pride and self-worth increased across the course of 6 months. This LC acknowledged that the job has *“given me my value my worth”*. Similarly, a PR stated that *“I built on that a lot as well. My personal opinion of myself”.* These participants recognized the significance of this role for their own growth and their understanding of who they are and what they can achieve. Every participant discussed some level of support they had received and how this helped them to develop their own skills and personal capabilities as outlined above.

Responses among the PRs and LCs differed to some extent. Generally, LCs appeared to have more confidence about their skills and in applying them to their role. For example, while during the 6-month interview one LC expressed that *“I’m very much more confident. I now know that I can do it”* a PR expressed that the role is *“helping me to develop my life confidence”.* While both the LCs and PRs credited the role to an increase in confidence, there appeared to be a difference in how much their confidence has developed. Leadership was also viewed quite differently across the PRs and LCs. One PR stated that *“I’ve never been interested in being a leader or anything else. It’s not for me”* while conversely a life coach stated that they *“automatically gravitate to them [leadership positions] because I see that’s where I can make a difference”.*

#### Theme 3: constraints to wellbeing

A mutual point of discussion among the LCs and PRs, was the impact that their sense of cultural and community responsibility (code 3.1) had on their wellbeing. While only two LCs and one PR expressed feeling the weight of cultural and community responsibility during the baseline interview, most LCs and PRs seemed to feel the impact by the 6-month interview (code 3.5). During the baseline interviews, the two participants who discussed this also discussed their fears regarding how to best support their community and predicted how this might negatively impact their wellbeing. During the 6-month interview, PRs and LCs described a sense of compassion fatigue. For example, one life coach said that *“you[‘re] really stressed for these people when you get to know them”.*

Participants also discussed that the COVID-19 lockdowns in Melbourne negatively impacted their wellbeing which at times reduced their capacity to support their community. Participants expressed a shift in priorities from maintaining their cultural connection and community engagement to trying to survive and maintain their sanity during this challenging period. Some LCs and PRs also discussed the challenges of connecting with their culture and community more generally and the impact this had on their identity, sense of self, and overall wellbeing. The experiences of some participants can be linked to the ongoing impacts of colonization (code 3.2 and 3.6). For example, through engaging with community members, participants were reminded of their own experiences or of past colonial practices that continue to have an impact, such as the Stolen Generation. Namely, one participant said that *“I can empathize with the elders as well, just like the stolen gen stuff”.* This participant continued, saying that *“working in Indigenous organisation just constantly keeps you around your culture, constantly. We’re always looking…to make the wider community understand and accept”.* This participant demonstrates the protectiveness of engaging with one’s culture and community along with the importance of educating the wider community of how protective culture and community can be for Aboriginal people.

Across both the baseline (code 3.3) and 6-month (3.7) interviews, LCs and PRs discussed how they would have appreciated more support from the organisation. During the baseline interviews, participants generally provided suggestions for how they could be supported with one participant stating that they “*still need some guidance”*. In contrast, during the 6-month interviews, participants discussed how they would have liked to be better supported with one participant saying that *“When I reflect, I wasn’t really supported”.* Another focus of the 6-month interviews was on workplace employment challenges (code 3.8). Participants discussed the impact these challenges had on their ability to do their jobs and on their mental health. For example, one participant reflected that “*the lack of clarity on what work we were actually doing made it really hard to just do it well”*.

Overall, both the LCs and PRs discussed more generally about some of their experiences of poor emotional wellbeing (codes 3.4 and 3.9). There were some participants who spoke during the baseline interview about the challenges they were facing prior to attaining their role at AHV. However, there was more discussion regarding overall wellbeing during the 6-month interviews. Some participants found that their emotional wellbeing was negatively impacted across the course of the 6-months. However, this was generally related to the COVID-19 lockdowns. For example, one participant reflected, *“I’m quite a free spirit and being restricted was hard”* while another lamented, “*a bit more stressed because I feel like I’m probably not doing…the best that I can”.*

#### Theme 4: community engagement and cultural connection

During the baseline interviews, participants explained why the domains of community and culture are important to them (code 4.1). While some participants shared more responses than others, each participant clearly identified at least one domain that was important to them. Some participants also identified areas for further learning. Participants most commonly expressed the importance of understanding their culture and community. Other responses included *“family connection”* and *“passing on that knowledge to our younger children”.* During the baseline interviews, most participants expressed having already gained a deeper understanding of community needs (code 4.2 e.g., *“seeing, seeing people grow, actually physically seeing [it]”*) or an eagerness to increase this understanding (e.g., *“getting more of an understanding of what the community needs”*). However, by the 6-month interview this understanding appeared to have extensively increased among all LCs and PRs. Participants discussed how important it is for them to engage with and support their community (code 4.5) and how much they enjoy it. One life coach said that *“I love working here. I love working with the clients. I love watching the goals that they kick”.* Many participants also received feedback from the tenants they were working with, which further increased their sense of pride. One participant said that *“hearing how happy and proud they [tenants] are in themselves. Its contagious”.* These participants acknowledged the significance of supporting their community and how this impacted on their own wellbeing (codes 4.1 and 4.2).

By the baseline interview, participants were already beginning to see the benefits of community interaction (code 4.3) both for themselves (e.g., *“how to act within a community”)* and for the community itself (e.g., *“be out there in my community…and be generous with Aboriginal housing”).* These benefits were further acknowledged during the 6-month interviews (code 4.6). Similarly, while participants generally acknowledged having a sense of community engagement and cultural connection during the baseline interviews (code 4.4), this understanding markedly grew over the course of 6 months (code 4.7). For example, at baseline one participant said that this role has helped in *“understanding a bit more about my culture”*. Whereas at the 6-month interviews, one life coach said that *“my understanding as well of like the needs of community is a lot clearer…my connection grows that way”.* Participants also discussed their increased awareness of cultural events and that engaging with both Aboriginal tenants and colleagues helped them to enhance their cultural understanding. Finally, participants expressed being grateful for the sense of community that their colleagues provided and acknowledged that the stressful nature of their jobs were made easier with the support of their team (code 4.8).

#### Theme 5: employment facilitators

At baseline, all LCs and PRs made a clear link between their current role and an increase in either their future career opportunities (code 5.1) or in their overall work potential. By the 6-month interviews, this link became clearer to participants (code 5.3). Participants expressed a sense of gratitude for the opportunity to work at AHV and the realization of future potential (code 5.4) in other domains of their lives including financial independence and a sense of empowerment. Discussion with the LCs also extended to feeling inspired to pursue further study or career planning opportunities beyond their current role (code 5.5). However, this sentiment was only discussed during the 6-month interviews when participants appeared to start thinking about their careers beyond AHV. Through these discussions, it appeared that employment at AHV allowed participants the freedom to contemplate future employment and education. While the LCs appeared to be more inspired to make large steps in their careers (e.g., *“more future planning of where I want to be”)* as compared with the PRs, several PRs acknowledged the significance of this role in terms of *“getting your foot in the door [and] working with community”*. Despite not expressing a sense of being inspired to seek future employment, by the 6-month interview two PR had attained new jobs outside of AHV. These PRs were making active and successful steps in achieving future work. The participants who had gained employment by the 6-month interview expressed that employment at AHV helped them to attain the new role. Other participants who were still employed at AHV discussed the opportunities that might arise in the future because of their current role. Overall, the most notable changes from baseline to 6-months were that participants became more confident and aware of their own potential within the workforce. For example, one participant demonstrated that having a job at AHV granted them the opportunity to “*think what do I really want to do?”*.

## Discussion

Through this study, we evaluated the experiences of life coaches (LCs and peer researchers (PRs) employed to deliver an Aboriginal and Torres Strait Islander community-based research project. Specifically, we aimed to explore potential changes to participants access to strengths and resources (i.e., resilience) through the collection of largely qualitative data with trends confirmed by quantitative data. The quantitative results revealed non-significant improvements in access to strengths and resources among the LCs and PRs and therefore suggests our hypothesis was not met. However, these quantitative results also showed that there was a moderate to large increase in access to strengths and resources based on scores on the ARRQ from baseline to 6-months. These results are encouraging as demonstrated through an average score increase in both sub-scales. While these increases were non-significant, given the small sample size, this is not surprising. Further, given our results revealed a large effect size (which as per scholars Sulivan and Feinn, importantly demonstrates the magnitude of the change) our results prove promising [45]. We argue that a larger sample size or a longer study design may have elicited significant results.

Our results demonstrated that Aboriginal staff employed as LCs and PRs reported increases in access to strengths and resources across a 6-month period. The MTAL project was not necessarily designed to enhance resiliency outcomes of the PRs and LCs, however despite the COVID-19 lockdowns and the added challenges this had on participants’ jobs, it appears PRs and LCs were able to build on the capacity to access strengths and resources. Similarly, Gee and colleagues found that involvement in Aboriginal-designed empowerment pilot programs improved ARRQ scores among those involved in the programs [22]. Their results revealed that the programs significantly enhanced participants’ access to personal, relationship, community and cultural strengths and resources from baseline to week 12 and week 16 respectively.

Similarly to the current study, Schultz and Cairney explored the benefits of employment [49]. They revealed that employment in Indigenous land management programs can lead to empowerment, greater engagement with healthy behaviors, and enhanced knowledge. Together with our study, these findings provide evidence related to the protective role of employment and how it may contribute to improvements in empowerment, resilience, knowledge, and health behaviors. However, while literature has explored wellbeing outcomes among Aboriginal and Torres Strait Islander Peoples employed as researchers, our study is one of the first to specifically explore the relationship between employment and resilience among this cohort. In particular, our study utilizes the ARRQ (an Indigenous-designed survey) to capture changes in resiliency patterns and support our qualitative findings on resiliency. In contrast, previous literature has focused on either qualitative or quantitative data rather than both. Finally, our study differs to previous work by capturing changes in resiliency across a period of 6 months. In doing so, we were able to explore how 6-months of community-based employment might impact an individual’s sense of resilience.

Our qualitative interviews also suggested overall positive impact on participants’ aspirations, cultural and community engagement, wellbeing, empowerment, relationships, and opportunity to lead. In line with our hypotheses, participants generally reflected that being employed positively impacted their social, emotional, cultural, and financial wellbeing. In addition to the positive impacts that employment elicited for participants, we also suggest that involvement in the peer researcher and life coaching training at the beginning of employment may have had positive impacts. This training was an important component of this employment and aimed to provide the LCs and PRs with the skills to not only complete their role but also to look after themselves. In particular, there was one component on self-care, which may have supported the wellbeing and resilience of the PRs and LCs throughout their employment.

Participants also revealed some of the challenges of their employment. A major theme emerging from the qualitative interviews was the importance of aspirations, with participants emphasizing 6-month improvements in their employment and financial goals. In an investigation in state-representative samples in Australia and the USA, Doery and colleagues noted significantly lower rates of independent income and paid employment for Indigenous compared to non-Indigenous young adults [16]. The current findings suggest community research programs may offer a means of addressing inequitable access to income and employment experienced by Indigenous Peoples.

Most participants reflected on how helpful it was to discuss their own goals during the interview process. Goal setting has been shown to be beneficial for well-being [50] and was reported to increase across the 6-month employment. Several participants reflected that they spent much of their job speaking to community members about their goals and that the interview provided the PRs and LCs with an opportunity to do the same. All participants set goals during the baseline interview and had achieved at least one of these goals by the 6-month interview. In addition, many participants wanted to set themselves additional goals and discuss how they could achieve more. Despite presenting the participants’ goals across specific domains, their goals were diverse. This diversity demonstrates that researchers, organisations, and health care services need to work with individuals to allow them to voice their goals and to identify the support they might require to achieve these goals. We know that the goals and aspirations of First Nations Peoples continue to be ignored and that this likely plays an integral part in the mistrust that First Nations peoples have in the health care system and in research [51, 52]. Our study provided participants with the space to share, reflect, and make progress towards their goals.

Additional benefits were also noted on participants’ sense of cultural and community engagement, sense of empowerment, their skill development, and confidence in their own capabilities. While benefits were seen among both LCs and PRs some differences between the two groups were also seen, particularly regarding sense of confidence and leadership. Differences may be due to several factors including the age of participants (the average age of PRs was 27 years, and the average age of LCs was 37.5 years) and the differences in responsibility and commitment required of the two roles.

Many of these concepts (i.e., sense of cultural and community engagement, sense of empowerment) can be understood within the SEWB framework. Our study contributes to our overall understanding of how we can best support the SEWB of Aboriginal and Torres Strait Islander Peoples. Many of these benefits identified by the current participants are reflected in existing studies which have evaluated the Family Wellbeing program (FWB). The FWB is a large community-based project that has been running within Aboriginal and Torres Strait Islander communities throughout Australia since 1993 [53, 54]. While most studies explore the impact of this program on the health and wellbeing of program participants and community members, limited research has also explored the impact on the researchers. For example, McCalman and colleagues demonstrated how involvement with the program supported Aboriginal researchers’ sense of community [55]. Cath Brown also reflected on her involvement in the same program as a research officer [56]. Reflections included a greater sense of empowerment, skill development, the importance of support from team members, a sense of hope regarding the program, and a greater sense of their own strengths. Many of these reflections are echoed in the responses of the participants in the current study.

Some of the challenges that participants reflected on included a sense of cultural responsibility, the ongoing impact of colonization, workplace challenges, and inadequate support. Many of these challenges have been cited as common risk factors for poor mental health among Aboriginal and Torres Strait Islander Peoples [12, 23, 57]. Similar challenges have also been experienced by other researchers. For example, Haynes and colleagues revealed that Aboriginal community researchers faced challenges related to interpersonal relationships and cultural demands [7]. Some community researchers also felt the project outcomes were somewhat limited and they were not able to achieve their goals within the life of the project [7]. Knowing the consequences of these challenges and the risks they pose, we need to develop our understanding of these challenges, the impact they have on overall wellbeing, and work alongside communities to mitigate the consequences.

In acknowledging the burdens of community and cultural responsibility and an inadequacy of support that some participants noted, it is also important to reflect on the possible iatrogenic effect (risk associated with interventions) of community-based research. While employment as either a peer researcher or life coach was intended to enhance community and cultural engagement, develop skills, support goal progression and achievement, and develop resilience and empowerment, there is also a possibility that employment may have caused harm. While the qualitative data suggested that the positive impacts of their employment were far greater than the challenges and negative impacts, it is important to acknowledge the possible harms of community-based project in developing future research protocols. Despite the current researchers taking significant steps to address cultural safety through strategies such as consultation with community members and cultural awareness training, there remained some challenges and constraints to wellbeing among participants. While there is a large body of literature that has explored the iatrogenic effect of different evaluations and interventions, there remains a paucity of literature that has explored this effect within First Nations community-based research. We suggest that future research further consider this effect and reflect on strategies to minimize the potential negative consequences of the employment of First Nations People in research. While there are clear benefits of community-based research, genuine and significant steps to protect those involved in the research are required.

In reflecting on these findings, we must also consider some of the study limitations. Firstly, while the research team consisted of males and females, and Indigenous and non-Indigenous researchers, all interviews were conducted by the same female, non-Indigenous interviewer. It is possible that the interviewers cultural background and/or gender may have limited participants’ honesty and openness in sharing their experiences, which may have consequently reduced the richness of data. Therefore, involvement of an Aboriginal or Torres Strait Islander researcher in data collection and the use of Indigenous research methodologies and techniques (for example Yarning) would likely create a more culturally safe space for participants and elicit richer data collection. Further, it might have been beneficial for the interviewer to be someone who the participants felt familiar and comfortable with. It is important to further acknowledge that within our Protocol [35], Yarning was identified as a technique that would be utilized throughout the interviews but was ultimately not used in the study. This decision was made upon reflection and discussion among the authors and with reference to current literature [55]. Such literature suggests that Yarning should be implemented under the leadership of an Indigenous person [55]. Another point of difference between the current study and our Protocol was the sample size and quantitative analysis. In our Protocol we outlined that the sample would include 10 PRs, five LCs, and five additional staff, whereas the study included five PRs, five LCs, and zero additional staff. We also outlined that a repeated measures Analysis of Variance would be conducted rather than the Paired Samples *t* tests that were ultimately implemented. The decision to changes analyses was made based on the reduced sample size. Overall, any changes that were made between the publication of our Protocol and this study (of which there were minimal) were done following discussion between co-authors, with consideration of the evidence, and based on best practice.

Similarly, each interview was audio-recorded and while this allowed the researchers to capture entire interviews, this may have further reduced participants’ willingness to share. This may be particularly relevant for the current participants, given research demonstrates that First Nations People have both historically and more recently been exploited in research [58]. However, to build rapport and trust with participants, the interviewer attended two weeks of cultural training with the participants and implemented culturally safe practices throughout the interview and research project. This encouraged the researcher to create a comfortable environment and establish trust and good rapport. Further, Indigenous researchers contributed to the analysis and reporting of the data and overall development of this paper. In addition to the recommended ethical guidelines, future researchers may wish to utilize storyboards as a data collection approach as this requires active participation and greater engagement.

We also acknowledge that the small sample size reduced the statistical power for the quantitative analysis. Further, given that purposive sampling was utilised, whereby a specific sample of people was selected and that the sample was small, participants may have had concerns regarding their anonymity. These possible concerns may have led participants to limit their responses. However, the Plain Language Statements informed participants of possible confidentiality and anonymity issues. Further, we removed obvious identifying information and only included short segments of quotes within the results section to reduce the likelihood of identification either by the participant themselves or by community members.

A final limitation is that while participants identified with various Aboriginal and Torres Strait Islander communities around Australia, they may not be representative of the wider population. This study explored the experiences of ten Aboriginal and Torres Strait Islander Peoples who participated in a specific project. It is likely that the impact of employment in a different community-based project will elicit different results. However, there was consensus among all participants that their employment was beneficial across a range of social, emotional, cultural, and economic domains. These limitations notwithstanding, we have demonstrated that beyond the research outcomes and the community, Aboriginal and Torres Strait Islander Peoples employed to complete research can also benefit from community-based research.

## Conclusions

The current study revealed that employment in a community-based project had a predominately positive impact on the wellbeing, relationships, resilience, opportunity to lead, aspirations, goal setting skills, connection to culture and community, and empowerment of Aboriginal and Torres Strait Islander PRs and LCs. Our results further demonstrated that the LCs and PRs experienced changes in their personal, relationship, community and cultural strengths and resources across the course of 6 months. Overall, we conclude that while some participants experienced some levels of stress because of the role and COVID-19 lockdowns, most participants acknowledged an improvement in their overall emotional wellbeing. Some participants accredited this to their employment, while others felt that there was a more indirect link, suggesting that having a consistent job helped them to feel more stable in other areas of their lives. Consistently, participants acknowledged the benefits to their own sense of cultural and community understanding through supporting and engaging with their community. Similarly, participants reflected on the value of setting goals and in reflecting on progress made towards these goals.

Our quantitative results, which demonstrated moderate to large improvements in access to strengths and resources by the LCs and PRs supported the qualitative results in that participants were able to access resources (e.g., community, culture, and relationships with themselves and others) to support their overall wellbeing. We acknowledge that while our study importantly revealed the impact of employment and goal setting on several domains of wellbeing, additional research is required. We encourage future researchers to collect data at more than two timepoints and more deeply explore what the term wellbeing means to participants. We also suggest that it may be useful for future research to employ larger sample sizes and longitudinal research designs to offer support for external validity. Finally, we also encourage policymakers to continue to implement community-based research that is culturally appropriate, empowering, and supports the self-determination priorities of participants, community members, and the researchers. Employment may be a useful tool to enhance wellbeing outcomes among Aboriginal and Torres Strait Islander Peoples. However, it should be combined with culturally relevant support and adherence to the goals and aspirations of those employed. With the combination of relevant support and employment, the wellbeing of Aboriginal and Torres Strait Islander Peoples could be improved.

### Electronic supplementary material

Below is the link to the electronic supplementary material.

Supplementary Material 1

## Data Availability

Due to the sensitive nature of this research, the nature of qualitative interviews, and the sample size (10 participants) individual privacy could be compromised if we share the raw data publicly. However, the data can be made available upon request from the corresponding author (Elizabeth Doery).
