# Supplementary material for: Impact of community-based employment on Aboriginal and Torres Strait Islander wellbeing, aspirations, and resilience
Source: BMC Public Health. 2024 Feb 16;24:497. doi: 10.1186/s12889-024-17909-z (PMC10870455; doi:10.1186/s12889-024-17909-z)
Supplement: Supplementary file 1 — Supplementary Material 1 [file 12889_2024_17909_MOESM1_ESM.docx]

**Supplementary Material**

**Additional File: One**

Demographic questions asked to life coaches and peer researchers during the baseline and 6-month interviews.

1. Which of the following cultural groups do you identify with?
2. Aboriginal
3. Torres Strait Islander
4. First Nations
5. First Peoples
6. Australian South Sea Islander
7. Non-Indigenous People
8. Other…………………….
9. What is your age now? ………………….
10. What is your current marital status?
11. Single, never married
12. Engaged
13. Married
14. Living with partner, not married
15. In a relationship, not married
16. Divorced
17. Separated
18. Widowed
19. Prefer not to answer
20. Which of the following best describes where you currently live?
21. Owned house/apartment
22. Rented house/apartment
23. University/college residential accommodation
24. Staying with friends temporarily
25. Refuge/temporary accommodation
26. Hotel/motel/caravan
27. No fixed address - currently on extended period of travel
28. Other (please describe)
29. Prefer not to answer
30. What is the highest level of secondary education or qualification you have received?
31. Never went to school
32. Attended primary school but did not finish
33. finished primary school
34. Year 7
35. Year 8
36. Year 9
37. Year 10
38. Year 11
39. Year 12
40. What is the highest level of tertiary education or qualification you have received?
    1. Certificate I/II
    2. Certificate III/IV (including trade certificate)
    3. Advanced diploma
    4. Bachelor degree (with or without honours)
    5. Graduate diploma/graduate certificate
    6. Post-graduate degree
41. What were your sources of income over the 6 months before joining this project?
42. Paid employment
43. Parental allowance. Please describe
44. Government allowance. Please describe
45. Other – Please describe
46. Prefer not to answer
47. Are you currently looking for work?
    1. Yes
    2. No
    3. Prefer not to answer
48. If any, what are the difficulties/problems you have had getting a job?
49. Transport problems or too far to travel
50. No jobs to match skills
51. No jobs in local area
52. Insufficient training
53. Ill health or disability
54. Discrimination because you are Aboriginal/Torres Strait Islander/Aboriginal or Torres Strait Islander
55. Don’t have a driver’s license
56. Unable to find suitable child care
57. Have not had any difficulties to get a job
58. Other. Please specify……………………. ­­­­­­­­­­­­­______________

**Additional File: Two**

Open-ended questions asked to life coaches at baseline

- 1. How did you become involved in the More Than A Landlord (MTAL) project?
  2. How do you expect involvement in the MTAL project to impact your life in terms of…?
     1. Future employment
     2. Personal social and emotional wellbeing
     3. Your goals and aspirations
     4. Connection to community
  3. Can you describe your short (6 month) and long-term goals (12+ months) in terms of…?
     1. Health
     2. Financial
     3. Educational and employment
     4. Family
     5. Housing
  4. Do you think you have the skills to develop a plan to achieve your goals and aspirations?
  5. How do you expect the MTAL project will help you to achieve your goals?
  6. How would you like to be supported throughout the MTAL project to achieve your goals?
  7. How will the MTAL project help you to feel empowered?
  8. Do you think that the MTAL project will provide you with a source of strength and resilience?
  9. How do you expect the MTAL project will impact your connection to culture and community?
  10. What exposure do you have to any leadership positions within your community?
  11. What aspects of your culture, traditions or lore are important to you?
  12. Do you feel strongly connected to your community?

**Additional File: Three**

Open-ended questions asked to peer researchers at baseline

1. Why did you become involved in the More Than A Landlord (MTAL) project?
   1. How did you find the peer researcher training experience?
2. How do you expect involvement in the MTAL project to impact your life in terms of…?
   - 1. Future employment
     2. Personal social and emotional wellbeing
     3. Your goals and aspirations
     4. Connection to community
3. Can you describe your short (6 month) and long-term goals (12+ months) in terms of…?
   - 1. Health
     2. Financial
     3. Educational and employment
     4. Family
     5. Housing
4. Do you think you have the skills to develop a plan to achieve your goals and aspirations?
5. How do you expect the MTAL project will help you to achieve your goals?
6. How would you like to be supported throughout the MTAL project to achieve your goals?
7. How will the MTAL project help you to feel empowered?
8. Do you think that the MTAL project will provide you with a source of strength and resilience?
9. How do you expect the MTAL project will impact your connection to culture and community?
10. What exposure do you have to any leadership positions within your community?
11. What aspects of your culture and traditional law are important to you?
12. Do you feel strongly connected to your community?

**Additional File: Four**

Open-ended questions asked to life coaches at 6-month interview

1. How has your experience with the MTAL project been so far?
2. What has encouraged you to stay involved in the MTAL project?
3. How many hours did you expect to work per week over the last 6 months?
4. How many hours did work per week over the last 6 months?
5. How has involvement in the MTAL project impacted your life in terms of…?
   - 1. Future employment
     2. Personal social and emotional wellbeing
     3. Your goals and aspirations
     4. Connection to community
6. Have any of your goals changed from the baseline interview?
   1. If so, how have they changed?
7. Which goals that you set out at 6/12 months ago have you successfully achieved?
8. Are you proud of yourself for achieving your goals?
9. Do you think the MTAL project developed your skills to plan and achieve your goals and aspirations?
10. How has the MTAL project supported you to plan and achieve your goals and aspirations?
11. How could the MTAL project better support you in achieving your goals and aspirations?
12. How has the MTAL project helped you to feel empowered?
13. Do you think that the MTAL project has provided you with a source of strength and resilience?
14. How has the MTAL project impacted your connection to culture and community?
15. Has the MTAL project provided you with exposure to any leadership positions within your community?
16. Do you feel more strongly connected to your community?

**Additional File: Five**

Open-ended questions asked to life coaches at 6-month interview

1. How has your experience with the MTAL project been so far?
2. What has encouraged you to stay involved in the MTAL project?
3. How many hours did you expect to work per week over the last 6 months?
4. How many hours did work per week over the last 6 months?
5. How has involvement in the MTAL project impacted your life in terms of…?
   - 1. Future employment
     2. Personal social and emotional wellbeing
     3. Your goals and aspirations
     4. Connection to community
6. Have any of your goals changed from the baseline interview?
   1. If so, how have they changed?
7. Which goals that you set out at 6/12 months ago have you been able to achieve or make progress towards?
8. How do you feel about achieving your goals?
9. Do you think the MTAL project developed your skills to plan and achieve your goals and aspirations?
10. How has the MTAL project supported you to plan and achieve your goals and aspirations?
11. How could the MTAL project better support you in achieving your goals and aspirations?
12. How has the MTAL project helped you to feel empowered?
13. Do you think that the MTAL project has provided you with a source of strength and resilience?
14. How has the MTAL project impacted your connection to culture and community?
15. Has this MTAL project provided you with exposure to any leadership positions within your community?
16. Do you feel more strongly connected to your community?

**Additional File: Six**

Survey used at baseline and 6-month interviews

1. **Participant ID:**
2. **Today’s date:**
3. **Gender:**

**The ARRQ**

Aboriginal Resilience and Recovery Questionnaire (ARRQ) [22, 40].

These strength-based questions ask how you would describe yourself, and the way you are with your family, community and culture I would like you to respond to them with one of five possible responses. The possible responses are: not at all (1), a little (2), some-what (3), a fair bit (4), a lot (5).

|  |  | Not at  All  **1** | A  little  **2** | Some-what  **3** | A fair bit  **4** | A  Lot  **5** |
| --- | --- | --- | --- | --- | --- | --- |
| 1 | I am proud to be Aboriginal or Torres Strait Islander |  |  |  |  |  |
| 2 | Being Aboriginal or Torres Strait Islander is an important part of who I am |  |  |  |  |  |
| 3 | I am able to maintain my Aboriginal or Torres Strait Islander identity, values and beliefs |  |  |  |  |  |
| 4 | In my community I have opportunities to develop skills (e.g., job skills or skills to care for others) |  |  |  |  |  |
| 5 | In my community I have opportunities to further my education |  |  |  |  |  |
| 6 | I have opportunities to work in my life, keep busy and stay involved |  |  |  |  |  |
| 7 | I am able to overcome most of my problems by working together with my friends and family |  |  |  |  |  |
| 8 | What happens to me in the future depends most of all on the support of my friends and family |  |  |  |  |  |
| 9 | I feel supported by my friends/mob |  |  |  |  |  |
| 10 | I can talk about my problems with family or friends |  |  |  |  |  |
| 11 | I have family that love me even when I muck up |  |  |  |  |  |
| 12 | In my family we can talk with each other about most things |  |  |  |  |  |
| 13 | In my everyday life I have role models that I look up to |  |  |  |  |  |
| 14 | In my everyday life I have people who listen to me and believe in me |  |  |  |  |  |
| 15 | There are people in my life that I have close, secure relationships with |  |  |  |  |  |
| 16 | I can turn to my partner or someone close to me for support and understanding |  |  |  |  |  |
| 17 | I feel safe when I am with my partner or those closest to me |  |  |  |  |  |
| 18 | I feel safe when I am with my family |  |  |  |  |  |
| 19 | I feel safe in my community |  |  |  |  |  |
| 20 | I feel safe in the broader society outside my community |  |  |  |  |  |
| 21 | I have a safe place to go to where I can heal |  |  |  |  |  |
| 22 | I feel pride in my achievements |  |  |  |  |  |
| 23 | I am ok with myself as I am now |  |  |  |  |  |
| 24 | When I experience setbacks, I don’t give up |  |  |  |  |  |
| 25 | I usually finish what I start |  |  |  |  |  |
| 26 | There is meaning in what I do in my daily life |  |  |  |  |  |
| 27 | I have things in my life that I’m passionate about |  |  |  |  |  |
| 28 | I can trust myself to make the right choice |  |  |  |  |  |
| 29 | Despite any bad experiences in the past, I am able to trust most people |  |  |  |  |  |
| 30 | I am aware of my strengths and weaknesses |  |  |  |  |  |
| 31 | I try to understand why things happen to me |  |  |  |  |  |
| 32 | I choose not to blame other people for the decisions I make |  |  |  |  |  |
| 33 | I am responsible for my own happiness |  |  |  |  |  |
| 34 | I can talk about how I feel when I get upset |  |  |  |  |  |
| 35 | I am able to face problems without gambling, using drugs or alcohol, or harming others |  |  |  |  |  |
| 36 | I can handle painful or upsetting emotions such as anger and sadness |  |  |  |  |  |
| 37 | On a daily basis things happen that make me happy |  |  |  |  |  |
| 38 | I feel content with my life |  |  |  |  |  |
| 39 | I feel compassion for the pain others feel |  |  |  |  |  |
| 40 | I am able to forgive myself and others |  |  |  |  |  |
| 41 | I am able to deal with most problems that occur in my life |  |  |  |  |  |
| 42 | When changes occur in my life, I can usually find ways to adapt |  |  |  |  |  |
| 43 | Overall, I feel like I have control over my life |  |  |  |  |  |
| 44 | What happens to me in the future depends most of all on me |  |  |  |  |  |
| 45 | I take positive action to try and solve problems |  |  |  |  |  |
| 46 | When I have a problem, I make plans about how to deal with it |  |  |  |  |  |
| 47 | I spend time helping others in my community |  |  |  |  |  |
| 48 | I know where to go in my community for help |  |  |  |  |  |
| 49 | I feel like I belong in my community |  |  |  |  |  |
| 50 | I am treated fairly in my community |  |  |  |  |  |
| 51 | I participate in cultural practices that give me peace (such as going out bush, ceremony, community cultural events) |  |  |  |  |  |
| 52 | Spirituality is a source of strength for me |  |  |  |  |  |
| 53 | When stressed I am able to take time to care for myself (e.g., time alone, relaxation) |  |  |  |  |  |
| 54 | I am able to accept difficult things that have happened in my past |  |  |  |  |  |
| 55 | I have opportunities to tell my story and make sense of things that have happened to me |  |  |  |  |  |
| 56 | I am able to have a laugh even when things are difficult |  |  |  |  |  |
| 57 | I use art, music, sports or similar activities as a way to express myself |  |  |  |  |  |
| 58 | I have the skills to be confident in both Indigenous and non-Indigenous communities |  |  |  |  |  |
| 59 | I find it easy to get along well with people |  |  |  |  |  |
| 60 | I feel confident in socialising with others around me |  |  |  |  |  |
| 61 | I speak an Aboriginal or Torres Strait Islander language(s) |  |  |  |  |  |
